# Trimetallic Single-Atom Alloys: Active Site Synergy or Most Reactive Atom Dominance? The Case of RhPtCu(111)

**DOI:** 10.1021/acs.jpcc.6c03621

**Published:** 2026-08-13

**Authors:** Vinita Lal, E. Charles H. Sykes

**Affiliations:** Department of Chemistry, 1810 Tufts University, Medford, Massachusetts 02155, United States

## Abstract

Elucidating how different atomically dispersed dopant atoms interact and control reaction pathways is central to the rational design of trimetallic single-atom alloys (SAAs). Here, we investigate CH_3__r_I decomposition on Cu(111), PtCu(111), RhCu(111), and RhPtCu(111) trimetallic SAAs in order to elucidate how coexisting dopant sites influence C–H activation, hydrogenation, and C–C coupling pathways. Temperature-programmed desorption measurements show distinct reactivity of the two bimetallic systems: PtCu(111) promotes C–C coupling to yield ethene and the methane produced originates mostly from C–H activation and subsequent hydrogenation of methyl groups. In contrast, RhCu(111) strongly suppresses C–C coupling chemistry reducing the ethene yield and produces methane mostly via hydrogenation by existing surface H. When Rh and Pt are codeposited to form a RhPtCu(111) SAA, the resulting reactivity and selectivity are not the sum of the two bimetallic SAAs. Instead, methane formation kinetics and product selectivity closely resemble bimetallic RhCu(111). Systematic variation of the Rh/(total Rh and Pt dopant) ratio shows that increasing Rh content progressively biases reactivity toward hydrogenation-dominated pathways while suppressing the C–C coupling pathway that leads to ethene formation. CO coadsorption experiments provide site-specific evidence for this behavior via selective blocking of Rh sites, which leads to a return to PtCu-like chemistry. Together, these results elucidate the reaction pathways on the RhPtCu(111) trimetallic SAA, showing that isolated Rh atoms act as dominant active sites that govern low-temperature reactivity and selectivity, thereby suppressing the C–C coupling facilitated by Pt sites at higher temperatures. This work demonstrates that RhPtCu(111) chemistry cannot be understood as linear combinations of their bimetallic counterparts, highlighting how one dopant atom can dominate the reaction pathways.

## Graphical abstract

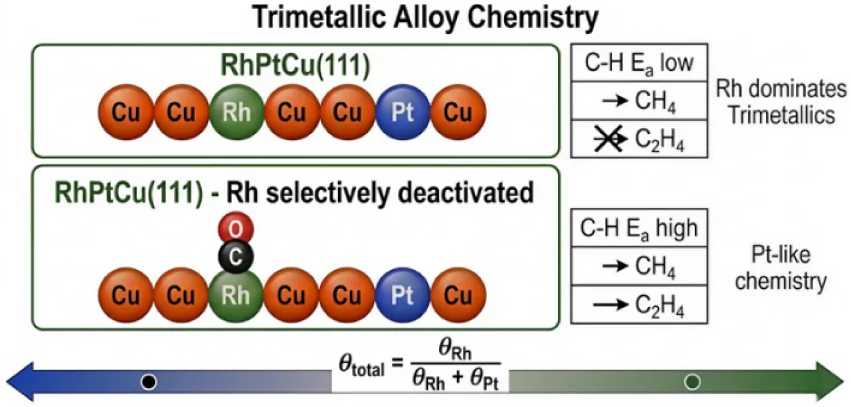

## Introduction

Single-atom alloys (SAAs), in which catalytically active metal dopants are atomically dispersed in the surface of a more inert metallic host, serve as model systems for studying structure–reactivity relationships that aid in the design of new heterogeneous catalysts.^1–3^ The geometric isolation of dopant atoms in SAAs suppresses ensemble effects thus enabling highly selective reaction pathways, while weak hybridization of the dopant atom with the host metal leads to free-atom-like electronic states that can provide unique reactivity.^4–7^ These features, and the simplicity of the active site, have enabled the design of supported SAA heterogeneous catalysts that exhibit exceptional performance in a wide range of reactions including hydrogenation,^8–23^ dehydrogenation,^24–35^ and selective oxidation.^36–40^

Moving forward, conventional SAAs rely on a single dopant element which may lack the ability to perform multistep transformations. As a result, reaction efficiency may be limited by the inability of a single site to simultaneously balance, for example, reactant activation, intermediate binding, and coupling requirements.^41–44^ This limitation motivates the extension of the SAA concept toward surfaces containing multiple chemically distinct dispersed single-atom active sites, where reactivity and selectivity may be enhanced as compared to the respective bimetallic systems.

In a related thrust, recent studies of trimetallic dual-atom site catalysts have demonstrated promising avenues for overcoming the constraints of single-dopant catalysts.^45–52^ Dual-atom alloys were first established using surface science model systems, where the controlled placement of two distinct dopant atoms as heteroatom pairs in a metal host enabled site-specific interrogation of cooperative reactivity. In these systems, heteroatom pairs composed of atoms with complementary chemical affinities can activate bonds or direct reaction pathways that are inaccessible to either SAA alone.^41,45,53,54^ While analogous effects have since been explored in supported nanoparticle and oxide-based catalysts, mechanistic interpretation in those materials is often complicated by structural heterogeneity, variable dopant nuclearity, and limited site-specific characterization.^50^ Metal-in-metal alloy surfaces therefore remain an essential platform for understanding the role of each dopant atom on chemical reactivity and selectivity with surface science techniques.^41,45,53–56^

The presence of multiple chemically distinct dispersed dopants does not necessarily guarantee cooperative or additive behavior. While adjacent dual-atom sites are designed to promote cooperative chemistry between paired heteroatoms, trimetallic SAAs containing spatially isolated dopant atoms raise a different mechanistic question: whether the dopants operate independently, act synergistically, or compete for the same reactant or surface intermediate. In the latter case, the more reactive site may dominate the reaction network and suppress pathways associated with the second dopant, making active-site dominance a possible outcome as opposed to a synergy that produces a product not formed by either bimetallic. Thus, while the identity of the dominant dopant will be system- and reaction specific, competition between isolated dopants for a common reactant or surface intermediate represents an important design consideration for trimetallic SAAs.

The literature on trimetallic SAAs (in which the two dopants are dispersed as atoms isolated from one another) is relatively sparse.^57,58^ It is therefore unclear to what extent the different isolated dopants operate independently, synergistically, or competitively, and how their distribution impacts the selectivity of competing pathways such as coupling, dehydrogenation, and hydrogenation. In the present work, we examine the reactivity of a RhPtCu(111) trimetallic SAA surface toward CH_3_I decomposition, with the goal of understanding how two chemically distinct dopant atoms separated from one another influence the reactivity of adsorbed alkyl groups. In the absence of a molecular beam that is required to activate e.g., CH_4_, CH_3_I is a widely used molecular probe for C–H activation and C–C coupling, because it readily dissociates on many metal surfaces at relatively low temperature to form adsorbed CH_3_ and I.^59–67^ The CH_3_ intermediate can then participate in hydrogenation to CH_4_ or in C–C coupling reactions, while adsorbed iodine is typically strongly bound and comparatively inert. Using temperature-programmed desorption (TPD), we compare the behavior of RhCu(111), PtCu(111), and RhPtCu(111) surfaces under the same conditions, and investigate the influence of dopant concentration and CO-mediated dopant deactivation effects on reaction pathways and product selectivity. We show that Rh atoms, even at low concentration, dominate the trimetallic SAA reactivity and selectivity by promoting both C–H activation and hydrogenation to methane while suppressing the C–C coupling that leads to ethene formation enabled by Pt atoms in Cu(111). In support of the proposed mechanisms, we demonstrate that the contribution of Rh to the trimetallic reactivity can be suppressed by adsorption of CO which selectively deactivates the Rh sites. These findings provide insight into the roles of, and competition between, isolated dopants in trimetallic SAA surfaces. This type of fundamental understanding will underpin the design of multisite catalysts that enable more complex reactions to be performed selectively.

## Experimental Methods

All TPD experiments were carried out in an ultrahigh vacuum (UHV) chamber with base pressure ∼1 × 10^–10^ mbar. The Cu(111) crystal was mounted on a manipulator using 0.25 mm diameter Ta wires. The crystal was resistively heated via the Ta wires using a DC power supply and was cooled to 87 K via a liquid nitrogen cryostat. The crystal surface was cleaned with repeated cycles of Ar^+^ ion bombardment using a hot filament sputter source from RBD instruments and thermal annealing to 723 K. The temperature was measured by a K-type thermocouple welded to the back of the crystal. To prepare the SAA surfaces, the dopant atoms (Pt or Rh) were deposited by electron bombardment evaporation of high purity rods using an EFM 3 flux monitored evaporator from ScientaOmicron. Both metals were alloyed at 383 K. Pt was deposited at a 10 nA flux while Rh was deposited at a 5 nA flux. EFM calibration was performed by taking the ratio of the saturated CO peak from Cu(111) and the CO desorbed from dopant atoms, assuming a CO packing density of 0.52 ML on Cu(111) and 1 CO per dopant atom as described in previous studies.^32,59,67,68^ CO deactivation experiments were done by first dosing 20 L CO (saturated dose) at different temperatures (83 K–373 K) to selectively block Rh and Pt sites, followed by CH_3_I dosing at 83 K.

## Results and Discussion

We first examined whether Rh and Pt atoms remain isolated from one another and hence chemically distinct when codeposited in the Cu(111) surface. Figure 1 presents saturation CO-TPD spectra for RhPtCu(111), benchmarked against the RhCu(111) and PtCu(111) SAAs. Two well-resolved CO desorption features were observed on the RhPtCu(111) trimetallic surface corresponding to the characteristic desorption temperatures of CO from isolated Rh and Pt atoms in Cu(111).^32,63^ No additional desorption features were detected at low dopant coverages, indicating the absence of mixed Rh–Pt ensembles or metallic clusters. These observations are consistent with a surface in which Rh and Pt are predominantly present as isolated, chemically distinguishable single-atom sites in Cu(111). It is well-known that CO desorption is highly sensitive to the dopant identity, ensemble size and chemical environment,^7,41,61^ therefore, formation of a significant population of RhPt heterodimers, mixed ensembles, or metallic clusters can be ruled out because no additional CO desorption features are observed. Based on the dilute dopant concentrations, nearest-neighbor Rh–Pt pair formation would also be statistically rare for a random dopant distribution. Thus, while rare Rh–Pt nearest-neighbor pairs cannot be completely excluded, the CO-TPD data and dilute preparation conditions indicate that they do not constitute a significant fraction of the surface dopant population and are unlikely to dominate the observed CH_3_I reaction chemistry. Therefore, the reactivity trends discussed below for the trimetallic system are interpreted in terms of competition between accessible isolated Rh and Pt single-atom sites, rather than chemistry occurring primarily at Rh–Pt heterodimer sites.

**Figure 1:**
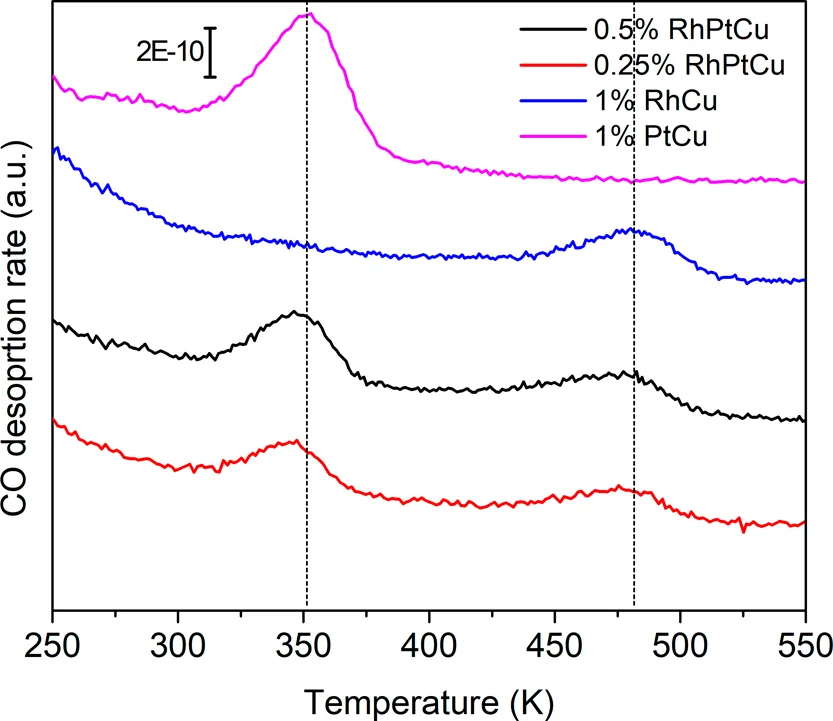
Saturation CO-TPD traces from Cu(111), PtCu(111), RhCu(111), and RhPtCu(111) surfaces following adsorption of 20 L CO at 83 K and linear heating at 1 K/s. Distinct desorption features corresponding to CO bound to isolated Pt and Rh atoms demonstrate atomic dispersion of dopant sites in the trimetallic alloy.

To first establish the reaction mechanisms exhibited by the individual SAAs, CH_3_I decomposition was examined on Cu(111), RhCu(111) and PtCu(111) under identical experimental conditions. In agreement with previous studies,^32,63^ CH_3_I undergoes facile C–I bond scission on Cu(111) to generate surface-bound methyl (CH_3_) groups, followed by the rate-limiting C–H activation step that produces methane (*m*/*z* = 16) via fast attachment of the cleaved H to another methyl group which desorbs spontaneously as methane. Accordingly, the methane evolution temperature serves as a sensitive reporter of the C–H activation barrier, with the desorption temperature following the trend Cu(111) > PtCu(111) > RhCu(111). Representative full TPD spectra for all products are provided in Figure S1.

On clean Cu(111), methane desorption occurs only at relatively high temperatures, reflecting the intrinsically high barrier for C–H activation on Cu surfaces.^32,69^ Methane formation is accompanied by a measurable ethene peak (*m*/*z* = 27), indicating that once C–H activation occurs, C–C coupling and β-hydride elimination occur leading to the formation of ethene as seen in Figure 2. Incorporation of isolated Pt atoms into Cu(111) lowers the temperature required for C–H activation while preserving access to coupling pathways, resulting in enhanced ethene formation relative to clean Cu(111) as seen in Figure 2. In contrast to PtCu, the RhCu(111) SAA exhibits markedly different selectivity, characterized by a pronounced low-temperature methane feature at 161 K attributed to hydrogenation of methyl groups with preadsorbed H from the chamber background. The RhCu(111) SAA also has a substantially reduced C–H activation temperature that leads to methane production (270 K), and near-complete suppression of ethene formation. The suppression of ethene formation occurs on RhCu because all the CH_3_ intermediates react and leave the surface as CH_4_ <300 K before ethene is formed on PtCu ∼320 K or Cu ∼390 K. It should be noted that, although methane and ethene remain the principal hydrocarbon products, consistent with previous studies,^32,63^ their desorption temperatures are slightly lower than the previously reported values characteristic of a slightly higher step-edge density on the present Cu(111) surface. Together, these SAA benchmark measurements establish the distinct and contrasting reactivity of pure Cu, PtCu and RhCu SAAs, providing a reference for interpreting the trimetallic chemistry.

**Figure 2:**
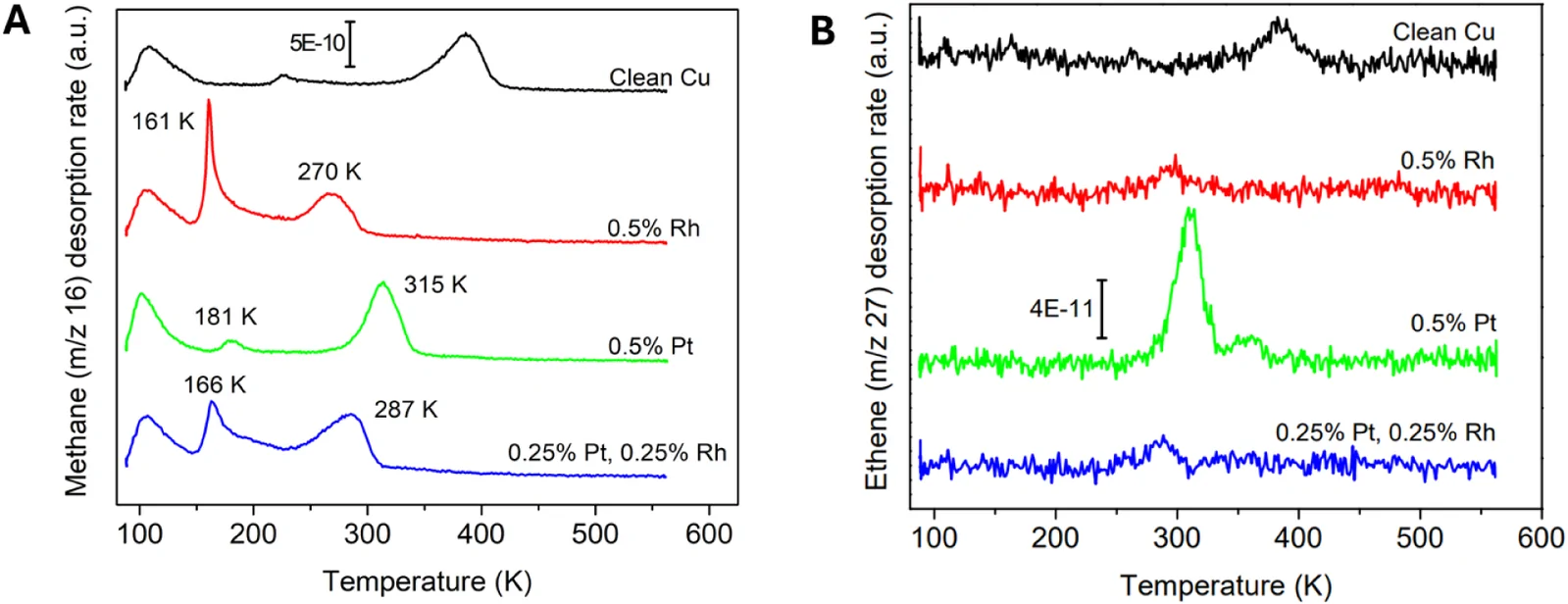
(A) Methane and (B) ethene desorption spectra from clean Cu(111), 0.5% RhCu(111), 0.5% PtCu(111) and 0.25% RhPtCu(111) following adsorption of 2 L CH_3_I at 83 K and linear heating of 1 K/s. Dopant coverages of the SAAs and the trimetallic are quoted as % of a monolayer.

We next examined CH_3_I decomposition on the RhPtCu (111) trimetallic SAA, initially with equal dopant concentrations (0.25% Rh, 0.25% Pt). Figure 2 directly compares methane and ethene desorption from clean Cu(111), the two SAAs and RhPtCu(111) under identical conditions. Detailed product traces are presented in Figure S2. Despite the presence of Pt single atoms, the methane desorption profile on RhPtCu(111) closely resembled that observed on RhCu(111). A pronounced low-temperature methane feature (166 K) was observed at essentially the same temperature (161 K) as on RhCu(111), indicating that Rh sites still facilitate hydrogenation of methyl groups with preadsorbed background hydrogen.

In addition, for the RhPtCu(111) trimetallic the higher-temperature methane feature associated with intrinsic C–H activation appeared only 17 K above that on RhCu(111) but 28 K lower than the PtCu(111) SAA. This behavior demonstrates that the kinetics of C–H activation in the trimetallic system is governed primarily by Rh rather than Pt or any synergy between the two dopants. Notably, no measurable ethene or higher hydrocarbons (Figure S2) were detected on the RhPtCu(111) trimetallic across the temperature range investigated, in stark contrast to PtCu(111), where C–C coupling and β-hydride elimination pathways were accessible. The absence of coupling products confirms that the trimetallic SAA surface does not exhibit a linear combination of Pt and Rh SAA behavior; instead, Rh dominates the reaction pathways by removing all the CH_3_ intermediates as CH_4_ and thereby suppressing the C–C coupling to ethene channel that occurs at higher temperature, even when an equal amount of Pt is present.

Approximate CH_4_ and C_2_H_4_ product yields are provided in Table S1 to compare the number of product molecules to the dopant atom coverage. The CH_4_ yield is similar on Cu(111) and all alloy surfaces, ∼0.013–0.014 ML, indicating that Rh and Pt primarily change the temperature and pathway of methane formation rather than the total methane yield. In contrast, C_2_H_4_ formation, which is roughly an order of magnitude lower than CH_4_, is more sensitive to dopant identity, increasing on PtCu(111) and decreasing on RhCu(111) and RhPtCu(111). Normalized to the total dopant coverage, the methyl-derived products correspond to ∼3–4 carbon containing products per dopant atom, suggesting that product formation is not limited to a single CH_3_ reaction event per dopant site.^32,63^

Figure 3 shows how Rh begins to dominate the reactivity of the trimetallic as it is added to a 0.5% PtCu alloy surface even at very low concentrations (0.03% monolayer Rh). Control experiments with pure Cu(111) and the 0.5% PtCu bimetallic are given on the left side of the figure. It can be seen that Pt enhances the selectivity of Cu to ethene, whereas addition of just 0.03% monolayer Rh reduces ethene selectivity, which continues to drop as more Rh is added until it reaches that of the 0.5% RhCu bimetallic.

**Figure 3:**
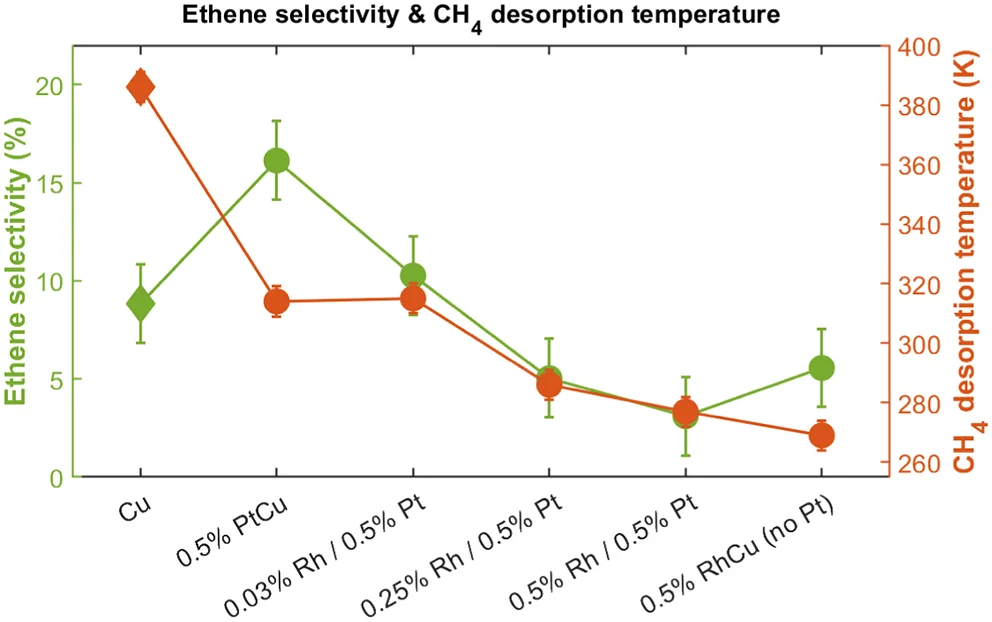
Ethene selectivity (green, left axis) and reactively formed CH_4_ desorption temperature (orange, right axis) as increasing amounts of Rh were alloyed in Cu while maintaining a constant Pt coverage of 0.5% monolayer. Clean Cu and Pt-free RhCu are included as controls. While Pt alone increases selectivity to ethene, increasing Rh content suppressed the C–C coupling pathway that produced ethene and shifted CH_4_ desorption to lower temperature, consistent with Rh reducing the barrier for C–H activation and promoting methane formation at lower temperatures.

Methane desorption temperature showed a similar trend as seen on the right axis of Figure 3. On clean Cu, methane formation occurred at substantially higher temperature ∼390 K, consistent with the larger barrier associated with C–H activation on the undoped surface. Pt incorporation lowered the methane desorption temperature to ∼310 K. As Rh was added to 0.5% PtCu samples, the methane desorption temperature shifted further downward, reaching ∼270–280 K for Rh-rich compositions and the RhCu bimetallic thus outcompeting the ethene formation pathway. The origin of this Rh dominance likely reflects the ability of methyl intermediates to sample multiple surface sites during the TPD temperature ramp rather than remaining irreversibly trapped at the dopant site initially encountered. If CH_3_ species were permanently trapped at isolated Pt or Rh sites, the trimetallic reactivity would be expected to more closely resemble a linear combination of the PtCu(111) and RhCu(111) SAAs. Instead, the RhPtCu(111) surface exhibits Rh-like methane formation and suppressed ethene production, even when Pt and Rh are present in equal amounts. The approximate yields in Table S1 further support this interpretation because product formation is not limited to a single CH_3_-derived event per dopant atom. These results indicate that methyl intermediates are not permanently confined to their initial adsorption sites during heating, allowing them to access more reactive Rh sites, where low-temperature C–H activation and hydrogenation outcompete the Pt-mediated C–C coupling pathway that requires a higher temperature. While preferential incorporation of dopants near step edges could increase local proximity between dopants and methyl intermediates, the CO-TPD data indicate that appreciable Rh–Pt heterodimer or mixed-ensemble formation does not dominate the observed chemistry and that the dopants are still atomically dispersed due to their low surface coverage.

As established by the CO-TPD measurements shown in Figure 1, CO desorbs from Pt sites at substantially lower temperatures (∼350 K) than from Rh sites (∼475 K). This enables us to selectively block the Rh sites via CO adsorption (referred to as the Rh atoms being “corked” in Figure 4) while leaving Pt sites accessible over the temperature window relevant to CH_3_I reaction. Upon selective CO adsorption at Rh sites by dosing CO at higher temperature so it does not bind to Pt sites we find the low-temperature methane desorption peak arising from hydrogenation of methyl groups by background hydrogen on RhPtCu (111) was strongly attenuated. Significantly, the methane formed from C–H activation of surrounding methyl group shifted toward higher temperatures, approaching PtCu-like behavior, consistent with the Rh sites being deactivated by CO (Figure 4). Furthermore, ethene formation increased when Rh sites were deactivated demonstrating that the suppression of C–C coupling in the trimetallic system in the absence of CO originates from the presence of accessible Rh sites. Importantly, when both dopant species were deactivated with CO, no additional changes in methane or ethene desorption were observed relative to Rh-only deactivation because CO begins desorbing from Pt at ∼310 K enabling methyl groups access to the Pt sites for C–H activation and C–C coupling that leads to methane and ethene formation ∼320 K. These results support our finding that Rh dominates the trimetallic SAA chemistry because CO only begins desorbing from Rh at 430 K after all reactions are complete. Detailed CO-mediated deactivation TPD results are provided in Figure S4.

**Figure 4:**
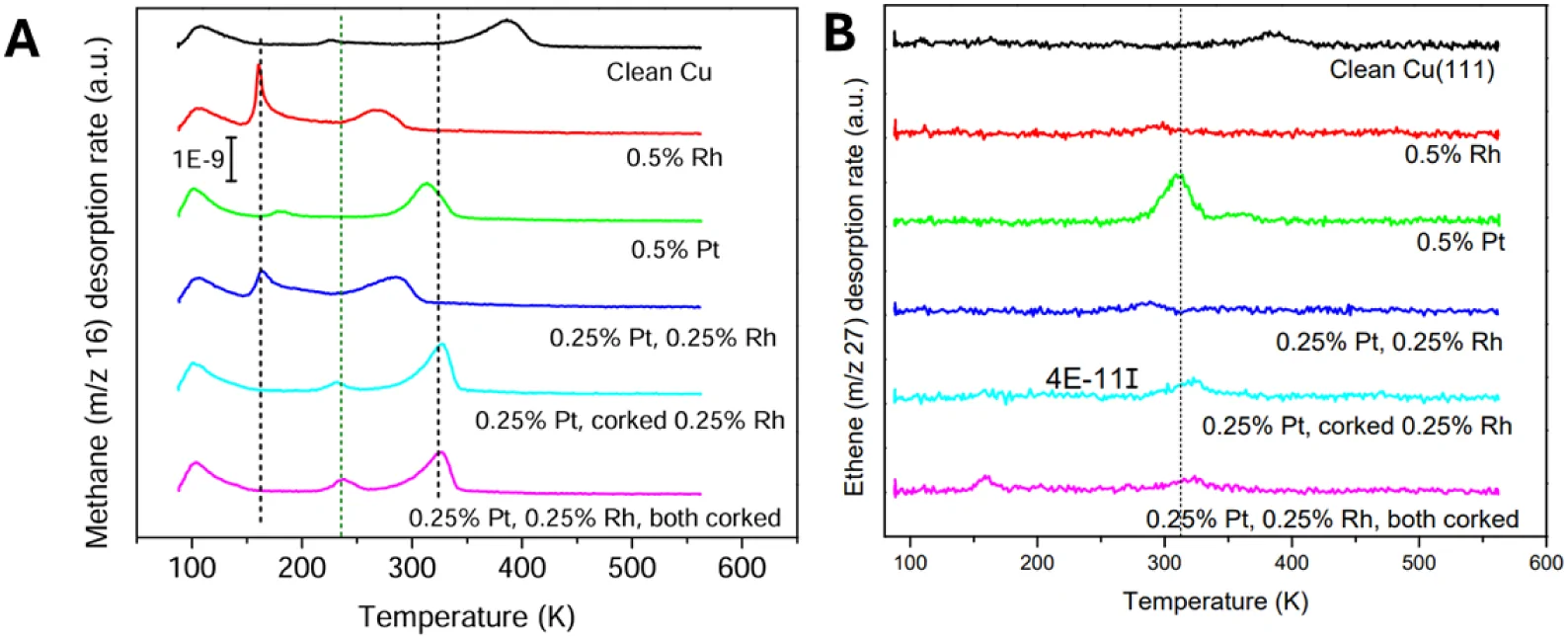
(A) Methane and (B) ethene desorption spectra from clean Cu(111), bimetallic systems and trimetallic system in the absence of CO, Rh sites with adsorbed CO, and both dopant sites with adsorbed CO following CH_3_I adsorption, illustrating site-specific control of reaction pathways. Surface coverages of dopant atoms are quoted as % monolayer.

## Conclusion

In this work, CH_3_I decomposition on Cu(111), RhCu(111), PtCu(111), and trimetallic RhPtCu(111) SAA surfaces was used to examine how multiple atomically dispersed dopants control alkyl group reaction pathways. Pure Cu(111) produced methane and ethene via high-temperature C–H activation in CH_3_ and C–C coupling to ethene, while the PtCu(111) SAA lowered the C–H activation barrier and enhanced C–C coupling. In contrast the RhCu(111) SAA promoted hydrogenation of methyl groups to methane, outcompeting C–C coupling chemistry. In contrast to a simple additive or synergistic combination of the bimetallic systems, the RhPtCu(111) trimetallic SAA exhibited Rh-dominated reactivity: methane formation kinetics and product selectivity closely resembled the RhCu(111) SAA, suppressing the C–C coupling pathway and shifting the surface toward hydrogenation-dominated pathways with increasing Rh content. CO site-blocking experiments confirmed that this behavior arose from accessible Rh sites, as selective deactivation of Rh shifted methane desorption toward Pt-like behavior and increased ethene formation. These results establish how isolated Rh atoms dominate the trimetallic reactivity by facilitating lower temperature C–H activation that leads to methane hence suppressing C–C coupling to ethene that requires a higher temperature. More broadly, this work demonstrates that the reactivity and selectivity of this particular trimetallic SAA is not a linear combination of its two bimetallic SAA parts. Rather, the reaction of methyl groups is dominated by Rh chemistry. This highlights the importance of site-specific chemistry in the rational design of multimetallic alloy catalysts and the need to identify dopant combinations that can act synergistically with each other rather than one that dominates the reaction pathway. For example, in a generic multistep reaction: A → B → C, if the first dopant catalyzes A → B but not B → C (where B is a surface-bound intermediate) and the second dopant catalyzes B → C but not A → B, one would expect the trimetallic SAA to show enhanced reactivity and selectivity to product C as compared to each bimetallic. We suggest that due to the many possible dopant combinations and different reactions one could use the existing SAA literature to predict promising dopant pairs to achieve such synergies.

## Supplementary Material



## Data Availability

Data will be made available on request.

## Notes

Additional TPD data illustrating suface composition- and dopant concentration-dependent CH_3_I reactivity, selective CO-induced deactivation of Rh and Pt sites, and estimated methane and ethene yields (PDF)
