# Supplementary material for: Trimetallic Single-Atom Alloys: Active Site Synergy or Most Reactive Atom Dominance? The Case of RhPtCu(111)
Source: J Phys Chem C Nanomater Interfaces. 2026 Aug 13;130(34):11961–7. doi: 10.1021/acs.jpcc.6c03621 (PMC13528300; doi:10.1021/acs.jpcc.6c03621)
Supplement: Supplementary file 1 [file jp6c03621_si_001.pdf]

Supporting Information

Trimetallic Single-Atom Alloys - Active Site Synergy or Most Reactive Atom Dominance? The case of RhPtCu(111)

Vinita Lal[1], E. Charles H. Sykes[1*]

[1]Department of Chemistry, Tufts University, Medford, Massachusetts 02155, United States

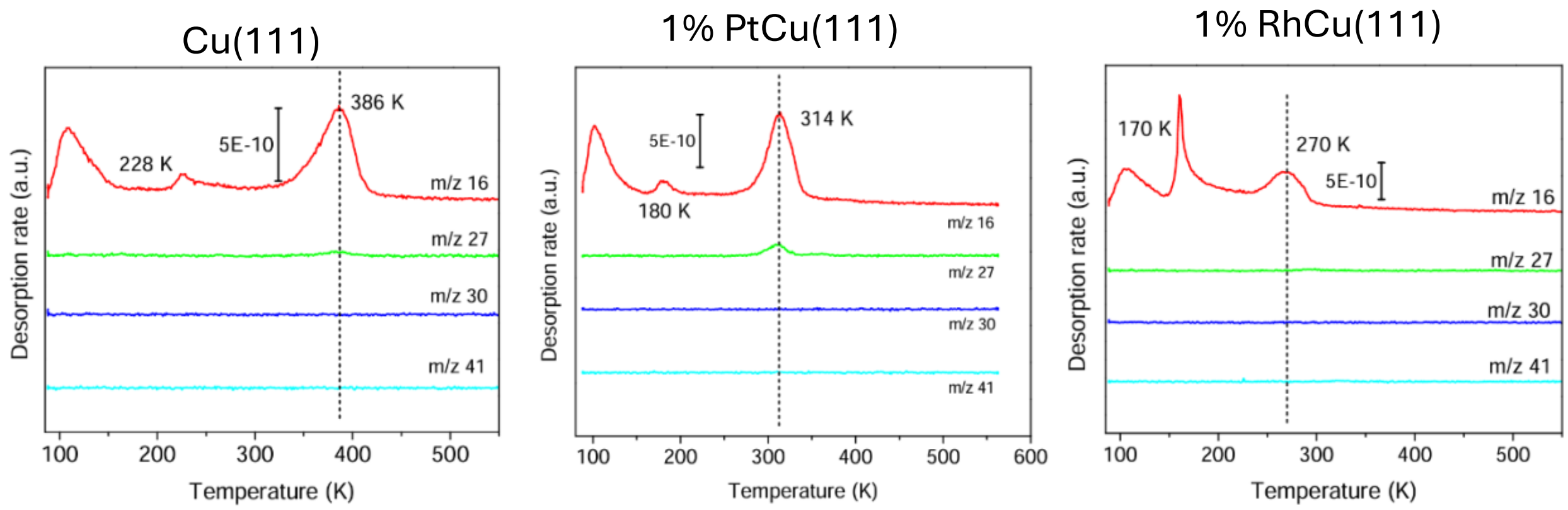

Figure S1: TPD spectra of products formed following CH_3I exposure to Cu(111), 1% PtCu(111), and 1% RhCu(111). 2 L CH_3I was dosed at 83 K followed by linear heating at 1 K/s. The traces correspond to methane (m/z 16), ethene (m/z 27), ethane (m/z 30) and propene (m/z 41).

Figure S1 presents representative TPD spectra used to establish baseline reactivity on Cu(111), PtCu(111), and RhCu(111) SAA surfaces, which are discussed in the main text (Figure 2). On Cu(111), methane formation occurred predominantly at high temperature, consistent with the low intrinsic activity of Cu(111) for C-H bond activation. Introduction of Pt lowered the methane desorption temperature associated with intrinsic C-H activation and enabled detectable ethene formation, indicating access to C-C coupling and β-hydride elimination pathways. In contrast, RhCu(111) exhibited a pronounced low-temperature methane feature attributed to hydrogenation from preadsorbed hydrogen from the UHV chamber background, along with a substantially reduced C-H activation temperature and suppression of ethene formation. These data establish distinct baseline behaviors against which trimetallic reactivity is compared in the main text. These results largely agree with previously published results, except that products desorb at lower temperatures on all three surfaces in the current study. This could be attributed to the Cu(111) surface having a higher step density.[1,2]

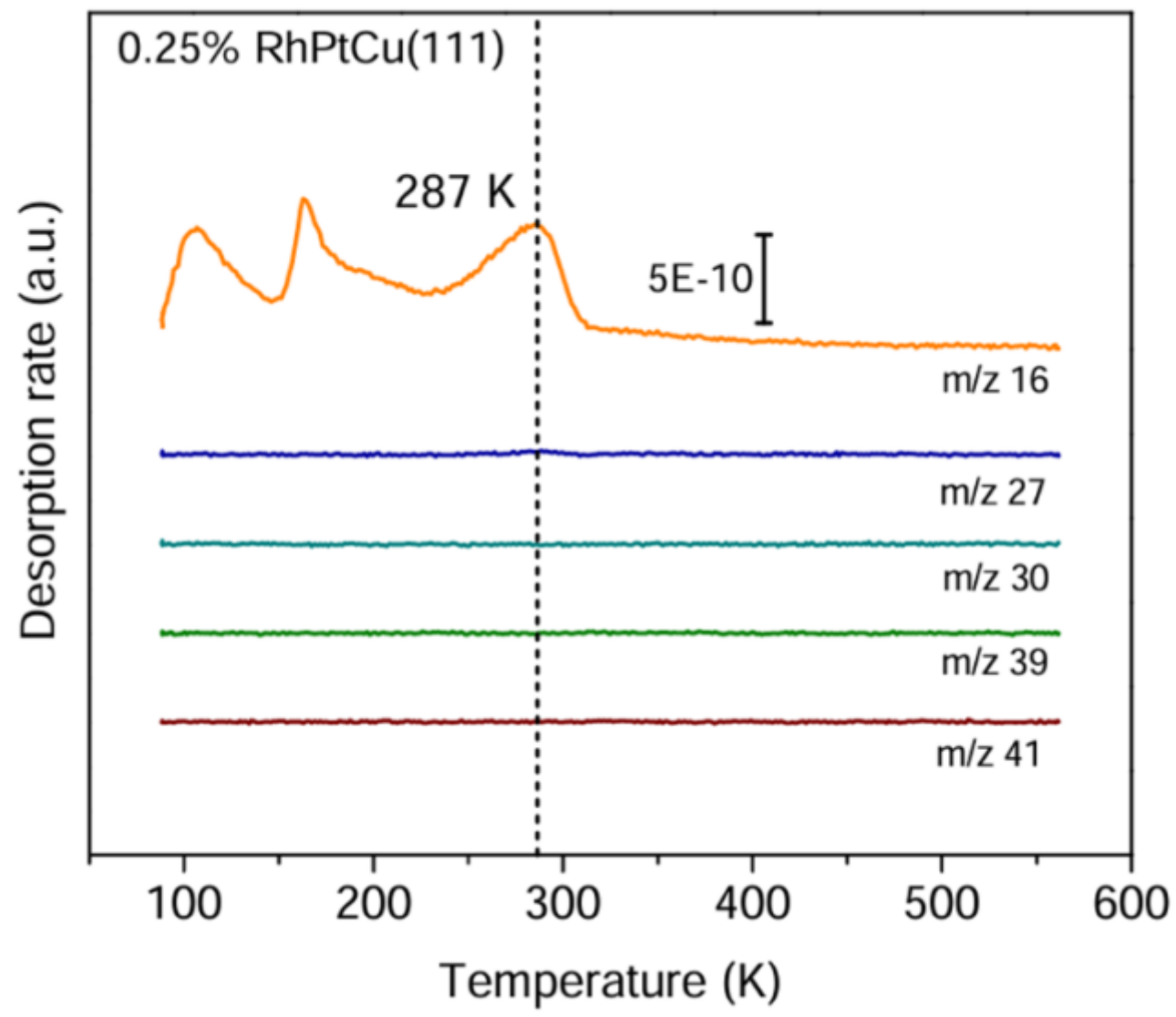

Figure S2: TPD spectra of products formed following CH_3I exposure to 0.25% RhPtCu(111). The trimetallic alloy surface comprised of 0.25% each of Rh and Pt deposited at 383 K. 2 L CH_3I was dosed at 83 K followed by linear heating at 1 K/s. The traces correspond to methane (m/z 16), ethene (m/z 27), ethane (m/z 30), 1,3-butadiene (m/z 39) and propene (m/z 41)

Figure S2 provides the complete product distribution for CH_3I decomposition on the RhPtCu(111) trimetallic SAA, supporting the methane and ethene data shown in the main text (Figure 2). In addition to methane and ethene, no significant formation of higher hydrocarbons was detected, indicating that coupling pathways remain strongly suppressed on the trimetallic surface under the conditions examined.

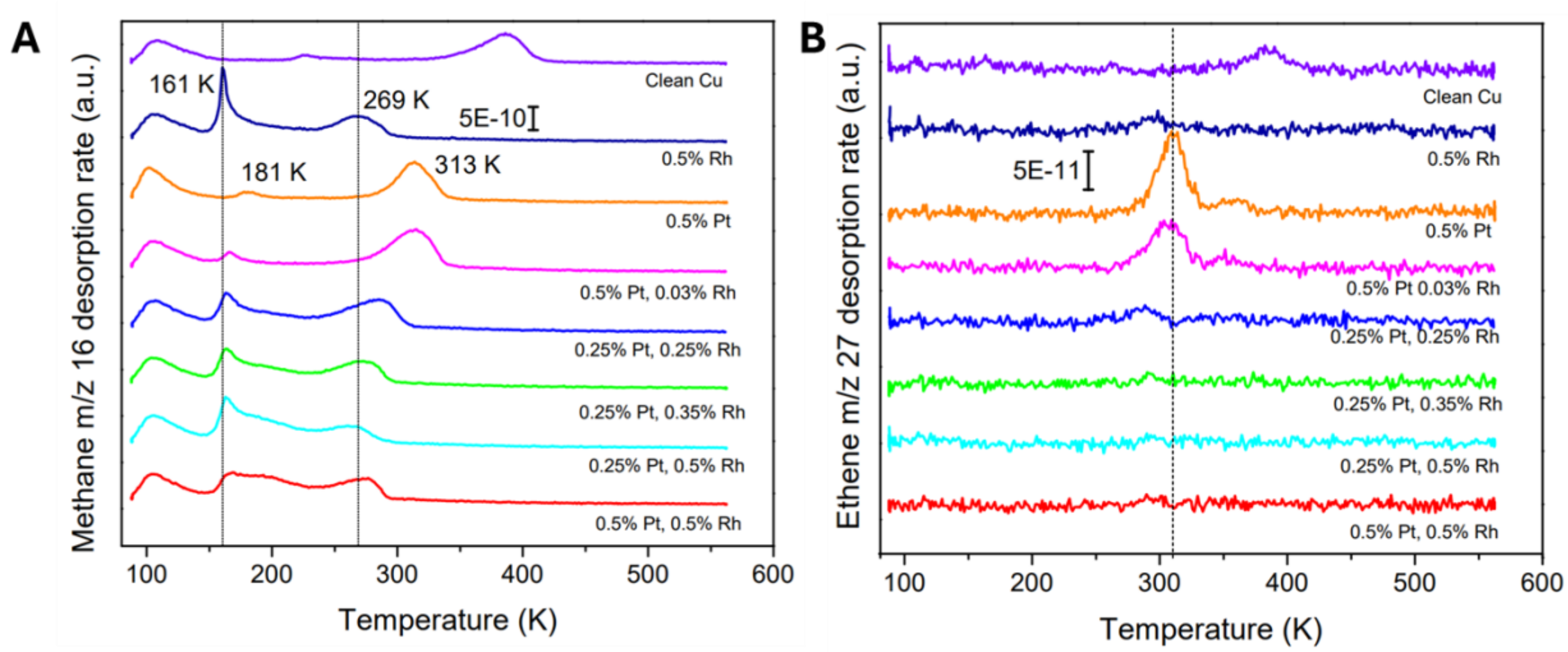

Figure S3: Detailed (A) Methane and (B) Ethene formation as a function of dopant concentration.

Figure S3 shows methane and ethene TPD traces for a series of mono-, bi-, and trimetallic surfaces. Two distinct methane features are observed: a low-temperature feature associated with hydrogenation from background adsorption and a higher-temperature feature corresponding to intrinsic C-H activation that forms methane. Increasing Rh content enhances the low-temperature hydrogenation feature and shifts the C-H activation feature to lower temperature, consistent with more facile C-H activation at Rh sites. Pt-rich compositions retain higher C-H activation temperatures than Rh and smaller hydrogenation features, highlighting the different influences of Rh and Pt on methane formation pathways. Ethene desorption is readily observed on PtCu(111) but is progressively suppressed upon introduction of Rh, even at very low Rh concentrations. Across the full compositional space examined, increasing Rh content leads to near-complete suppression of ethene formation.

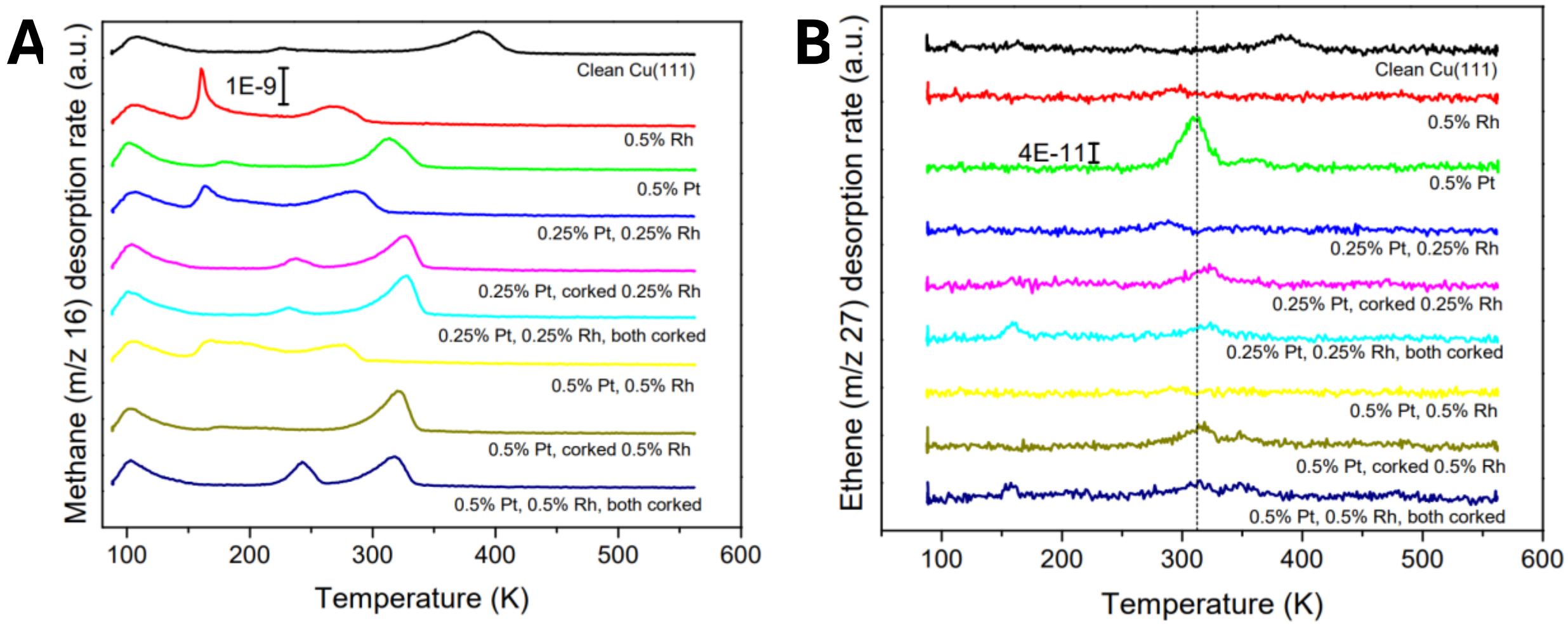

Figure S4: Methane (m/z 16) and ethene (m/z 27) TPD spectra from RhPtCu(111) surfaces on CO free, Rh deactivated, and both Rh and Pt deactivated with CO for different compositions

Figure S4 compares methane and ethene desorption from uncorked, Rh-corked, and fully corked trimetallic surfaces. Selective deactivating Rh sites with CO suppresses the low-temperature methane feature and shifts methane desorption toward higher temperatures, approaching PtCu-like behavior. When both dopant species are deactivated with CO, there is no significant change in methane formation because CO begins desorbing from Pt ~310 K. These results confirm that Rh sites dominate background hydrogen-mediated methane formation and C-H activation in the trimetallic system. Ethene formation is negligible on Rh-containing surfaces in the absence of CO, but increases upon selective Rh deactivation, confirming that suppression of C-C coupling originates from active Rh sites. When both dopants are covered with CO, again, no appreciable change is observed in ethene formation because CO begins desorbing from Pt ~310 K.

Surface	Dopant coverage (ML)	CH_4 yield (ML)	C_2H_4 yield (ML)	CH_4/dopant	C_2H_4/dopant	Carbon products/dopant
Cu(111)	-	0.0132	0.0013			-
0.5% RhCu(111)	0.005 Rh	0.0136	0.0008	2.7	0.16	3.03
0.5% PtCu(111)	0.005 Pt	0.0129	0.0025	2.6	0.49	3.56
0.25% Rh/ 0.25% PtCu(111)	0.0025 Rh 0.0025 Pt Total dopant = 0.005	0.0135	0.0006	2.7	0.12	2.94

Table S1: Approximate CH_4 and C_2H_4 product yields estimated from integrated TPD peak areas after baseline subtraction and sensitivity correction. Carbon products/dopant was calculated as $(Y_{\mathrm{CH4}} + 2Y_{\mathrm{C2H4}})/\theta_{\mathrm{dopant,total}}$.

References

(1) Hannagan, R. T.; Giannakakis, G.; Réocreux, R.; Schumann, J.; Finzel, J.; Wang, Y.; Michaelides, A.; Deshlahra, P.; Christopher, P.; Flytzani-Stephanopoulos, M.; Stamatakis, M.; Sykes, E. C. H. First-Principles Design of a Single-Atom-Alloy Propane Dehydrogenation Catalyst. *Science* 2021, *372* (6549), 1444–1447. https://doi.org/10.1126/science.abg8389

(2) Marcinkowski, M. D.; Darby, M. T.; Liu, J.; Wimble, J. M.; Lucci, F. R.; Lee, S.; Michaelides, A.; Flytzani-Stephanopoulos, M.; Stamatakis, M.; Sykes, E. C. H. Pt/Cu Single-Atom Alloys as Coke-Resistant Catalysts for Efficient C–H Activation. *Nat. Chem.* **2018**, *10* (3), 325–332. https://doi.org/10.1038/NCHEM.2915
